# Review of magnetic resonance-guided focused ultrasound in the treatment of uterine fibroids

**DOI:** 10.6061/clinics/2017(10)08

**Published:** 2017-10

**Authors:** Pedro Felipe Magalhães Peregrino, Marcos de Lorenzo Messina, Ricardo dos Santos Simões, José Maria Soares-Júnior, Edmund Chada Baracat

**Affiliations:** Disciplina de Ginecologia, Departamento de Obstetricia e Ginecologia, Hospital das Clinicas HCFMUSP, Faculdade de Medicina, Faculdade de Medicina, Universidade de Sao Paulo, Sao Paulo, SP, BR

**Keywords:** Leiomyoma, High-Intensity Focused Ultrasound Ablation, Ultrasonography, Magnetic Resonance Imaging, Menorrhagia

## Abstract

Uterine leiomyoma is the most frequently occurring solid pelvic tumor in women during the reproductive period. Magnetic resonance-guided high-intensity focused ultrasound is a promising technique for decreasing menorrhagia and dysmenorrhea in symptomatic women. The aim of this study is to review the role of Magnetic resonance-guided high-intensity focused ultrasound in the treatment of uterine fibroids in symptomatic patients. We performed a review of the MEDLINE and Cochrane databases up to April 2016. The analysis and data collection were performed using the following keywords: Leiomyoma, High-Intensity Focused Ultrasound Ablation, Ultrasonography, Magnetic Resonance Imaging, Menorrhagia. Two reviewers independently performed a quality assessment; when there was a disagreement, a third reviewer was consulted. Nineteen studies of Magnetic resonance-guided high-intensity focused ultrasound-treated fibroid patients were selected. The data indicated that tumor size was reduced and that symptoms were improved after treatment. There were few adverse effects, and they were not severe. Some studies have reported that in some cases, additional sessions of Magnetic resonance-guided high-intensity focused ultrasound or other interventions, such as myomectomy, uterine artery embolization or even hysterectomy, were necessary. This review suggests that Magnetic resonance-guided high-intensity focused ultrasound is a safe and effective technique. However, additional evidence from future studies will be required before the technique can be recommended as an alternative treatment for fibroids.

## INTRODUCTION

A uterine fibroid is a benign neoplasia originating from smooth muscle tissue. Uterine fibroids are classified as subserosal, intramural, and submucosal based on their location within the uterus. These different types of uterine fibroids may have diverse manifestations. The main symptoms are intense or prolonged menstrual bleeding (menorrhagia), dysmenorrhea, dyspareunia, adjacent organ compression (bladder and bowel), urinary incontinence, increased abdominal volume, infertility, and repeated miscarriages 1-3. Menorrhagia may cause anemia, which together with dysmenorrhea and dyspareunia, has a negative effect on the quality of life of these women. The increase in abdominal volume and the compression of adjacent organs may affect the intestinal and urinary habits of the patient, in addition to causing discomfort 3.

The classic treatments for fibroids include excision of the tumors and hysterectomy. However, such techniques may be very costly due to the length of surgery, increased bleeding risk, longer hospitalization, and possible postoperative complications. Therefore, researchers are developing new techniques such as magnetic resonance-guided high-intensity focused ultrasound (MRgFUS) 4-6.

MRgFUS is a minimally invasive therapy capable of producing necrosis by thermal coagulation of myometrial nodules at a precise location in the uterus 4. It is primarily used to decrease the volume of fibroids and to reduce complaints about symptoms. However, in many centers, MRgFUS is currently neither advocated nor established as an alternative therapy. Initial studies comprising various cohort sizes reported significant symptom improvement in response to MRgFUS. Even with partial results, MRgFUS has been shown to be an efficient and minimally invasive method for treating uterine fibroids 4-6.

Most of the studies in which MRgFUS has been used are case studies. Therefore, it is necessary to perform a systematic review of the studies to better assess MRgFUS as a therapeutic modality for uterine fibroids in symptomatic women during the reproductive period.

## METHODS

A review was conducted using articles in English, Spanish and Portuguese without publication year restriction up to April 2016 and retrieved from the MEDLINE and Cochrane databases. The following inclusion criteria were used: a) studies including patients of reproductive age with uterine fibroids; b) studies including patients with menorrhagic cycles, dysmenorrhea, and an increase in abdominal volume; c) the use of MRgFUS for the treatment of uterine fibroids. The exclusion criteria were as follows: a) studies of animal models; b) studies that included asymptomatic women; c) narrative reviews; d) case reports; e) cost assessments of therapeutic methods; f) comparisons of different techniques of the MRgFUS method; g) evaluation or study of the MRgFUS technique; h) studies with different protocols for the use of MRgFUS; i) studies that used a non-validated questionnaire.

**Figure 1:**
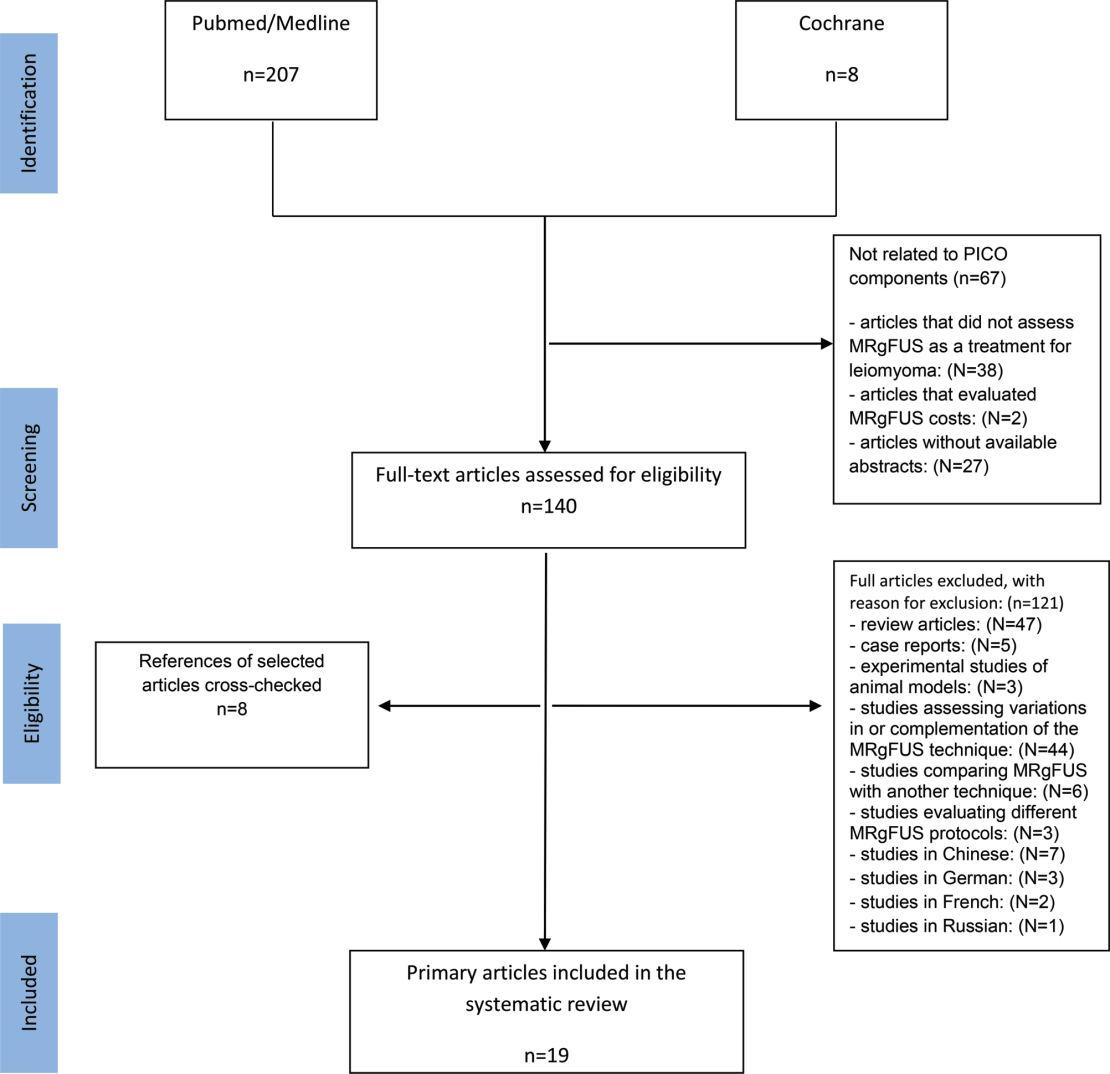
Flowchart: Selection of studies included in the review.

Articles were retrieved using the search strategies described in Box 1 and were grouped and structured according to the PICO strategy (the initials for “Patient”, “Intervention,” “Control” and “Outcome”). The search strategy is described in Figure 1.

Box 1 Databases and search strategies.

**Medline:** (Myoma OR Leiomyoma OR Fibroid OR Fibroids) AND (High-Intensity Focused Ultrasound Ablation OR High-Intensity Focused Ultrasound Ablation OR Ultrasonic Therapy OR HIFU OR MR-guided focused ultrasound OR MRgFUS). (N=207)

**Cϕchrane:** Leiomyoma AND High-Intensity Focused Ultrasound Ablation (N=8)

The references cited in the articles retrieved from the database were also evaluated. Systematic reviews and meta-analyses were consulted, but only the original articles were used. Two reviewers independently assessed the quality of the studies; when there was a disagreement, a third reviewer was consulted. The JADAD scale was used for the critical analysis of Randomized clinical trial.

## RESULTS

During the investigated period, a total of 207 articles were found in MEDLINE and 8 articles were found the in the Cochrane database (Figure 1). After the titles and abstracts were read and the eligibility criteria were applied, 188 articles were excluded (incomplete reviews, case reports, studies of therapy costs, studies in animal models, and studies of the comparisons of the different techniques of the MRgFUS method or studies with different protocols for the use of MRgFUS). The remaining 19 papers were included in this review 7-25.

The effects of MRgFUS on fibroid volume and on the patients’ symptoms and quality of life are summarized in Tables 1 and 2. In the 19 selected studies, patient age ranged from 32 to 50 years, and follow-up lasted from 3 to 36 months. This review comprised case studies and one clinical trial 24. Fibroid volume decreased in all the included studies; however, there was a discrepancy in the percentage reduction after MRgFUS. The decrease in fibroid volume ranged from 9,3% to 90% 11,19, as shown in Table 1.

Most studies used assessment questionnaires that have been validated in the literature. These assessments indicated that MRgFUS might improve the patients’ symptoms and quality of life 7-14,17-19,23-24. Some articles used only the symptom severity score (SSS), which is part of the Uterine Fibroid Symptom and Health-Related Quality of Life Questionnaire (UFS-QOL) to assess symptom improvement in patients. In the UFS-QOL, the SSS mainly analyzes symptoms such as menorrhagia and bulk-related symptoms. Three studies did not use validated questionnaires to evaluate quality of life or symptom improvement 14,20,25 or did not administer any specific questionnaires to evaluate patients’ symptoms and quality of life. These studies were included only in the analysis of the fibroid volume reduction. The decrease in the SSS ranged from 32% to 74%. Four studies used the complete UFS-QOL and reported increases in quality of life ranging from 20% to 47%. The only clinical trial included in this review analyzed 109 women with fibroids and showed a significant reduction of severe symptoms (51%) at 12 months of follow-up, which was statistically significant (*p*<0.001).

No severe complications were observed in the studies included in the analysis. The main complaints after MRgFUS were abdominal pain and lumbar pain, which improved with a mild analgesic. Two studies reported abdominal pain due to the accumulation of contrast material in the muscular and subcutaneous layers of the abdominal wall in 9% and 11% of the patients, respectively 7,13. In some cases, the necrotic material was expelled via the vagina after treatment over the subsequent menstrual cycles 11. The most severe complications occurred in a patient who developed deep vein thrombosis and required longer hospitalization 14 and in 2 patients who had endometritis with subsequent hysterectomy 15.

In some studies, new interventions were required because the patients’ symptoms persisted or worsened 8,12,15,17,21. For these reasons, Mindjuk et al. 8 reported a 12.7% rate of additional treatments such as hysterectomy, myomectomy, or embolization of uterine blood vessels. In the Funaki et al. 17 study, 1 patient underwent a hysterectomy, 5 patients underwent a myomectomy, and 5 others underwent a repeat MRgFUS after the primary treatment.

## DISCUSSION

The American College of Obstetricians and Gynecologists (ACOG) recommends that hysterectomies be included as an option for the treatment of benign conditions such as leiomyoma 26. Otherwise, myomectomy is the treatment of choice 1-3. However, recovery time, prolonged hospitalization, high rates of complications that may occur intraoperatively, and possible hysterectomies can be avoided. In this systematic review, MRgFUS was evaluated and found to be effective, promising, and safe for the treatment of benign uterine tumors. Hence, minimally invasive techniques such as MRgFUS may be an option, but further studies with a longer follow-up are necessary.

Since the introduction of MRgFUS and its approval by the FDA as a therapeutic modality (2004), several studies have been conducted and have reported symptom improvement in leiomyoma patients 6, as discussed in this review. The articles included in this study were mostly case series because it is difficult to conduct double-blind controlled studies with MRgFUS. This fact limits the design and performance of these studies. Additionally, the technique used with MRgFUS is very different from minimally invasive techniques such as embolization of uterine arteries 27.

Patient follow-up after MRgFUS lasted at least 3 months 7,10,13 and at most 36 months 14. Regardless of the variability in the length of follow-up, the studies under investigation reported reduced fibroid volume and a reduction in the patients’ symptoms. To assess the volume reduction, the nonperfused volume (NPV) after MRgFUS was considered. Only 9 of the 19 studies included in this review had an NPV over 50%. Although higher NPVs are associated with greater efficacy, symptom improvement was similar among the studies. It is possible that unevaluated uterine factors occurred after MRgFUS. Potential endometrial alterations may have a considerable impact on the uterus, primarily on a woman’s reproductive future. The studies included in this review did not evaluate this potential impact, i.e., they did not attempt to determine the feasibility of using the MRgFUS method in women with a desire to have children.

The differences in the previously mentioned results may be explained by the variety of fibroid types and the variation in signal intensity in the MRI T2 images soon after treatment 20,24. Tumors generating an MRI T2 hyposignal tend to exhibit higher necrosis rates and, consequently, larger reductions in fibroid volume. The reason for this is still unknown, but it seems to depend on both tumor volume and factors such as fibroid vascularization, intramural components, lesion density, and tumor-specific hormone expression 23.

Quality of life and obvious symptoms were assessed using the UFS-QOL. One potential limitation of the present study is that three of the included articles did not address variables of intense symptoms 15,21,25. A low SSS (a component of the UFS-QOL) indicates an improvement in symptoms, whereas an overall low UFS-QOL score indicates an enhancement of the patient’s quality of life. The articles in this review showed that the use of MRgFUS favors improvements in severe symptoms, as well as in the overall quality of life, despite the wide range of the follow-up time and the age of the study patients.

The SSS questionnaire primarily evaluates symptoms such as menorrhagia; therefore, its scores are more meaningful in studies of patients with intramural or submucosal fibroids. Taking this into consideration, Park et al. 10 studied 9 women with subserosal fibroids whose main complaints were mass effect and increased abdominal volume. They concluded that there was a significant reduction in volume and in mass effect (89%) but only a slight improvement in the SSS.

A better quality of life and improvements in bleeding and dysmenorrhea are also related to a larger NPV 11-13,19,23. Stewart et al. 23, whose study included more patients than any of the other studies in this review, analyzed 359 patients from all the clinical trials of MRgFUS in the treatment of fibroids and found that symptom improvement was significant. Furthermore, after a 24-month follow-up, they concluded that the SSS improvement was, on average, 6 points larger in the group of patients with the greater NPV. The relationship between NPV and symptom improvement may also be explained by the association of an MRI T2 hyposignal and a larger NPV, as found in some of the previously mentioned studies.

In all the studies included in the present analysis, the rates of adverse effects and complications following MRgFUS were low. The most common symptoms were abdominal pain, lumbar pain, first-degree burns, and light vaginal bleeding. The abdominal pain and lumbar pain were relieved with a common, mild analgesic. There was also pain caused by the accumulation of contrast material in the muscle and subcutaneous layers of the abdominal wall 7,13. However, neither hospitalization nor intervention were subsequently required to treat such complications. In general, tumors are highly vascularized and may undergo a parturition process after necrosis. In a few cases, the necrotic material was expelled via the vagina after treatment and disappeared after 4 menstrual cycles 11. The most severe complications were a case of deep vein thrombosis in 1 patient, which entailed a longer hospital stay 15, and 2 cases of endometritis requiring subsequent hysterectomy 16. Cases requiring intervention immediately after MRgFUS were rare. Hysterectomy, myomectomy, or uterine artery embolization was required for patients whose condition worsened or whose dysmenorrhea or hypermenorrhea relapsed.

To date, there are no randomized clinical trials that are suitable for inclusion in a meta-analysis, which would provide a more robust analysis. Our review suggests that MRgFUS is an effective and promising therapeutic technique for decreasing myoma volume and patients’ symptoms. However, further controlled and randomized studies will be required before MRgFUS can be recommended as an alternative fibroid treatment 7-25.

## AUTHOR CONTRIBUTIONS

Peregrino PF designed the study and was responsible for the analysis of data and manuscript writing. Messina ML designed the study, wrote and revised the manuscript. Simões RS designed the study and was responsible for the analysis of data and manuscript writing. Soares-Júnior JM designed the study and was responsible for the analysis of data and manuscript writing. Baracat EC designed the study and wrote and revised the manuscript.

## Figures and Tables

**Table 1:** Studies assessing the decrease in fibroid volume and the necrosis volume immediately after treatment in symptomatic women.

Author and Year of Publication	Patient Follow-up Time	Number of Patients	Age, Mean andStandard Deviation	Mean Reduction in Fibroid Volume	NPV Mean (%)
Park et al., 2014	3 months	79	43.6±4.4	23.1%	62.7±25.5
Kim et al., 2012	3 months	27	44.5±3.8	64.2%	64.2±19.9
Ikink et al., 2013	6 months	46	45.3±4.1	29%	40±22
LeBlang et al., 2010	6 months	80	44±3.2	31%	55±25
Morita et al., 2008	6 months	48	42.6±5.8	33%	60±18
Rabinovic et al., 2007	6 months	35	46.4±4.7	15%	31±23
Hindley et al., 2004	6 months	109	44.8±4.9	13.5%	25±6.0
Lenard et al., 2008	12 months	66	45.4±4.4	9.3%	16.3±13.3
Dobrotwir et al., 2012	12 months	74	42±7.0	38%	67±25
Wang et al., 2012*	24 months	78	38.2±6.4	90.1%	80±12
Funaki et al., 2009	24 months	91	40.4±4.6	39.5%	67±25
Kim et al., 2011	36 months	40	46±4.5	32%	32.1±6.2

NPV: nonperfused volume.

* Study of women with submucosal fibroids.

**Table 2:** Studies assessing the improvement of symptoms and the quality of life of symptomatic women.

Author and Year of Publication	Patient Follow-up Time	Number of Patients	Mean Age and Standard Deviation	Mean Reduction in SSS	Increase in the Overall UFS-QOL Score	Symptom Improvement	NPV Mean (%)
Park et al., 2014	3 months	79	43.6±4.4	35.6%	-	SIM	62.7±25.5
Park et al., 2012**	3 months	9	39.8±6.2	55.5%	-	SIM	66.9±10.6
Kim et al., 2012	3 months	27	44.5±3.8	35.8	-	SIM	64.2±19.9
Harding et al., 2008	6 months	102	45±4.8	44.8%	33.6%	SIM	-
Ikink et al., 2013	6 months	46	45.3±4.1	31.8%	19.5%	SIM	40±22
Mikami et al., 2008	6 months	48	45±5.2	-	-	SIM	47±13
Hindley et al., 2004	6 months	109	44.8±4.9	-	-	SIM	25±6.0
Lenard et al., 2008	12 months	66	45.4±4.4	38.8%	-	SIM	16.3±13.3
Dobrotwir et al., 2012	12 months	74	42±7.0	51%	-	SIM	67±25
Gorny et al., 2011	12 months	130	45.6±5.5	-	-	SIM	45.4±22.5
Stewart et al., 2006*	12 months	109	44.8±4.9	51%	-	SIM	-
Funaki et al., 2009	24 months	91	40.4±4.6	65.5%	-	SIM	67±25
Wang et al., 2012	24 months	78	38.2±6.4	-	31.25%	SIM	80±12
Stewart et al., 2007	24 months	359	45.4±5.0	35.6%	-	SIM	19.9±17.2/21.9±18.7
Mindjuk et al., 2014	24 months	252	42.1±6.9	70%	-	SIM	88.7±14.4
Kim et al., 2011	36 months	40	45.98±4.52	73.7%	47.4%	SIM	32.1±6.2

SSS: Symptom Severity Score.

* Clinical Trial.

** Study of women with pedunculated subserosal fibroids.
